# Selective Killing of BRCA2-Deficient Ovarian Cancer Cells via MRE11 Blockade

**DOI:** 10.3390/ijms241310966

**Published:** 2023-06-30

**Authors:** Adel Alblihy, Reem Ali, Mashael Algethami, Alison A. Ritchie, Ahmed Shoqafi, Shatha Alqahtani, Katia A. Mesquita, Michael S. Toss, Paloma Ordóñez-Morán, Jennie N. Jeyapalan, Lodewijk Dekker, Martina Salerno, Edgar Hartsuiker, Anna M. Grabowska, Emad A. Rakha, Nigel P. Mongan, Srinivasan Madhusudan

**Affiliations:** 1 Nottingham Biodiscovery Institute, School of Medicine, University of Nottingham, Nottingham NG7 3RD, UKmesquita.al0602@gmail.com (K.A.M.);; 2 Faculty of Medicine and Health Sciences, University of Nottingham, Sutton Bonington Campus, Sutton Bonington LE12 5RD, UK; 3 Nottingham Biodiscovery Institute, School of Pharmacy, University of Nottingham, Nottingham NG7 3RD, UK; 4 North West Cancer Research Institute, School of Medical and Health Sciences, Bangor University, Bangor LL57 2UW, UK; 5 Department of Pathology, Nottingham University Hospitals, City Campus, Nottingham NG5 1PB, UK; 6 Department of Oncology, Nottingham University Hospitals, Nottingham NG5 1PB, UK

**Keywords:** BRCA2, MRE11, synthetic lethality

## Abstract

The MRE11 nuclease is essential during DNA damage recognition, homologous recombination, and replication. BRCA2 plays important roles during homologous recombination and replication. Here, we show that effecting an MRE11 blockade using a prototypical inhibitor (Mirin) induces synthetic lethality (SL) in BRCA2-deficient ovarian cancer cells, HeLa cells, and 3D spheroids compared to BRCA2-proficient controls. Increased cytotoxicity was associated with double-strand break accumulation, S-phase cell cycle arrest, and increased apoptosis. An in silico analysis revealed Mirin docking onto the active site of MRE11. While Mirin sensitises DT40 *MRE11^+/^*^−^ cells to the Top1 poison SN-38, it does not sensitise nuclease-dead *MRE11* cells to this compound confirming that Mirin specifically inhibits Mre11 nuclease activity. MRE11 knockdown reduced cell viability in BRCA2-deficient PEO1 cells but not in BRCA2-proficient PEO4 cells. In a Mirin-resistant model, we show the downregulation of 53BP1 and DNA repair upregulation, leading to resistance, including in in vivo xenograft models. In a clinical cohort of human ovarian tumours, low levels of BRCA2 expression with high levels of MRE11 co-expression were linked with worse progression-free survival (PFS) (*p* = 0.005) and overall survival (OS) (*p* = 0.001). We conclude that MRE11 is an attractive SL target, and the pharmaceutical development of MRE11 inhibitors for precision oncology therapeutics may be of clinical benefit.

## 1. Introduction

The *BRCA2* tumour suppressor gene is essential for homologous recombination (HR) repair and replication fork stability maintenance [1]. Women carrying deleterious *BRCA2* germline mutations have a lifetime risk of up to 27% of developing ovarian cancers [2,3,4]. BRCA2-deficient, high-grade serous tumours are currently targeted in the clinic via synthetic lethality using poly ADP ribose polymerase (PARP) inhibitors (PARPis) (Niraparib, Olaparib, Rucaparib, or Talazoparib) [5,6]. In response to DNA damage, PARP1 binds to single-strand break repair (SSBR) intermediates and becomes activated, which in turn leads to the synthesis of PAR (poly-ADP-ribose) polymers. PARP1 auto-PARylation recruits other DNA repair factors at sites of DNA damage for the coordination of repair. PARPis not only block SSBR but PARP trapping disrupts replication fork progression, leading to double-strand breaks (DSBs). BRCA2-deficient tumours that are unable to repair DSBs accumulate lethal DSBs, which leads to selective cell death [5]. In clinical trials, PARPis have shown a response rate of up to 50% [6]. Non-responders (50%) have tumours that have intrinsic or acquired resistance to PARPi therapy [7,8,9]. Therefore, the development of alternative approaches to target DNA repair is desirable in BRCA2-germline-mutated ovarian cancers.

Emerging data suggest that additional factors within the DDR response could be promising synthetic lethality partners in BRCA2-deficient cells. RAD52 depletion in BRCA2-deficient cells decreased DSB-induced RAD51 foci formation and promoted extensive chromosome aberrations, especially chromatid-type aberrations [10]. RAD52 inhibitors that suppress the growth of BRCA2-deficient cells and inhibit RAD52-dependent single-strand annealing (SSA) have been developed [11]. In another study, the FDA-approved drug mitoxantrone was shown to specifically block RPA and RAD52 protein–protein interactions (PPIs) [12]. Mitoxantrone was selectively toxic in BRCA2-mutated ovarian cancer cells (PE01) in that study [12]. Although the mechanism of synthetic lethality is not fully known, the N-terminal domain of RAD52 may be essential for maintaining the cellular viability of BRCA1/2-deficient cells [13]. Moreover, RAD52 has been shown to compensate for the loss of BRCA2 function, interact with pCHK1, and maintain checkpoint control in BRCA2-deficient cells [14]. Interestingly, in clinical studies, BRCA2 mutation carriers inheriting the RAD52 S346X variant have a reduced risk of breast cancer [15,16,17]. Taken together, these data suggest that exploration of additional targets within the DNA damage response pathway (DDR) could be an attractive synthetic lethality approach.

The MRE11-RAD50-NBS1 (MRN) complex is critical for genomic stability [18,19,20]. MRN is required for the homologous recombination (HR), non-homologous end-joining (NHEJ), and microhomology-mediated end-joining (MMEJ) pathways. MRE11 has an N-terminal nuclease domain, a RAD50 binding motif, and a C-terminal DNA-binding domain. RAD50 has ATP and MRE11 binding sites. NBS1 has N-terminal BRCT-FHA domains and C-terminal MRE11- ATM (ataxia-telangiectasia mutated protein kinase) binding domains. The interactions of MRE11 with RAD50 and NBS1 promote MRN complex stability. The interactions of MRN with ATM and ATR (ataxia–telangiectasia-related protein kinase) promote DNA repair and cell cycle regulation [18,19,20].

MRE11 nuclease, a key component of the MRN complex, plays a critical role during the sensing, processing, and signalling of DNA double-strand breaks. MRE11 has endo- and exonuclease activities. Endonuclease activity is required during homologous recombination (HR), and the 3′-5′ exonuclease activity of MRE11 contributes to the processing of stalled replication forks [18,19,20]. Germline mutations in MRE11, although rare, can predispose an individual to cancer. Moreover, the somatic mutational spectra in MRE11, RAD50, and NBS1 can influence cancer phenotypes. BRCA2 and MRE11 interact with each other closely during replication fork stability maintenance [21]. In addition, PARP1 may promote the recruitment of MRE11 onto stalled replication forks [22]. In BRCA2-deficient cells, replication forks are extensively degraded by the MRE11 nuclease, leading to long stretches of single-strand DNA that may contribute to PARPi sensitivity [21,23,24].

We have recently shown that MRE11 upregulation is a frequent event in ovarian cancers and is associated with poor progression-free survival (PFS) and platinum resistance in patients [25]. Pre-clinically, MRE11 depletion or blockade by a small molecule inhibitor (Mirin) reversed platinum resistance [25]. Our data suggest that MRE11 is an attractive anti-cancer target in ovarian cancers. To validate this hypothesis, in the current study, we evaluated MRE11 blockade as a synthetic lethality strategy in BRCA2-deficient ovarian cancers.

## 2. Results

### 2.1. MRE11 Blockade Is Synthetically Lethal in BRCA2-Deficient Cancer Cells

BRCA2 protects stalled replication forks by stabilizing RAD51 filaments. MRE11 is not only essential for HR but is also required during the restart of stalled replication forks, prevents replication collapse during replication stress [26], and is involved in checkpoint activation [27]. We therefore tested whether a synthetic lethality relationship exists between BRCA2 and MRE11.

**Figure 1:**
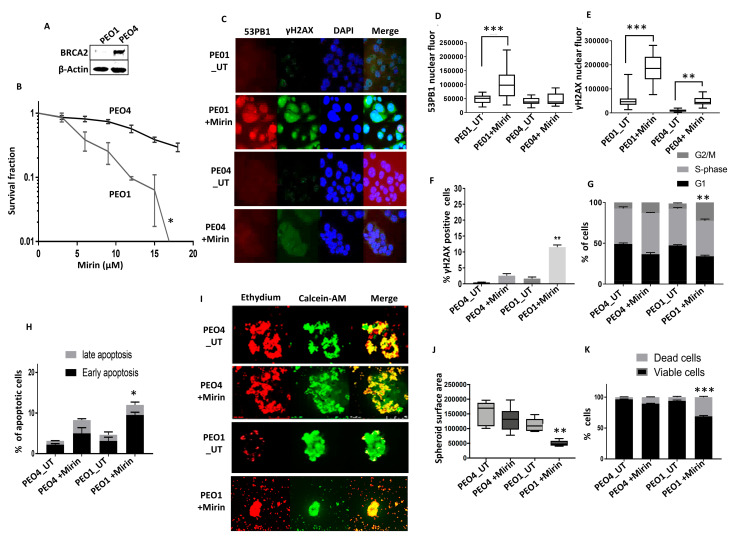
MRE11 blockade induces synthetic lethality in BRCA2-deficient ovarian cancer cells. (**A**) Western blot for BRCA2 and β-actin (control) protein levels in PEO1 and PE04 cells. (**B**) Mirin sensitivity, determined via clonogenic survival assay, in PEO1 and PEO4 cells. (**C**) Representative photomicrographic images for immunofluorescence staining of γH2AX, 53BP1, and DAPI (control) in PEO1 and PEO4 cells treated with Mirin (18 µM) for 24 h (magnification, ×20). (**D**) Quantification of 53BP1 nuclear fluorescence using ImageJ software (version 1.8.0_112) (UT = untreated; fluor = fluorescence). (**E**) Quantification of γH2AX nuclear fluorescence by ImageJ software (UT = untreated, flour = fluorescence). (**F**) γH2AX analysis via FACS. (**G**) Cell cycle analysis via flow cytometry in PEO4 cells treated with Mirin (18 µM) for 48 h (UT = untreated). (**H**) AnnexinV analysis to determine apoptotic cells in PEO4 cells treated with Mirin (18 µM) for 48 h (UT = untreated). (**I**) Representative photomicrographic images of PEO1 and PEO4 3D spheroids treated with Mirin (18 µM) (UT = untreated) (magnification, ×20). (**J**) Quantification of spheroid sizes using ImageJ software (UT = untreated). (**K**) Quantification of spheroids cell viability by flow cytometry (UT = untreated). ‘*’—*p* ≤ 0.05; ‘**’—*p* ≤ 0.01; ‘***’—*p* ≤ 0.001. All figures are representative of 3 or more experiments. Error bars represent standard errors of mean between experiments.

Mirin (Z-5-(4-hydroxybenzylidene)-2-imino-1,3-thiazolidin-4-one) is a small molecule inhibitor of MRE11 nuclease activity [28]. PEO1 is a BRCA2-germline-deficient ovarian cancer cell line derived from a patient with a poorly differentiated serous adenocarcinoma. The PEO4 cell line was derived from the same patient after the development of resistance to cisplatin chemotherapy. PEO4 is BRCA2-proficient, with the restoration of BRCA2 expression due to a secondary gene mutation. We first confirmed BRCA2 deficiency in the PEO1 cells compared to the PEO4 cells (Figure 1A). We then tested for Mirin cytotoxicity. As shown in Figure 1B, the PEO1 cells were sensitive to Mirin compared to the PEO4 cells. The increased sensitivity in the PEO1 cells was associated with DSB accumulation, which was assessed via increases in 53BP1 foci (Figure 1C,D) and γH2AX foci (Figure 1C,E), observed using immunofluorescence. A similar accumulation of γH2AX was also confirmed via FACS analysis (Figure 1F). The Mirin-induced DSB accumulation in PEO1 cells was associated with G2/M cell cycle arrest (Figure 1G) and increased apoptosis (Figure 1H). To mimic an in vivo system, we then generated 3D spheroids of these two ovarian cancer cell lines (Figure 1I). Mirin treatment significantly reduced the PEO1 spheroid size (Figure 1I,J) and increased dead cells (Figure 1K) compared to Mirin-treated PEO4 spheroids.

**Figure 2:**
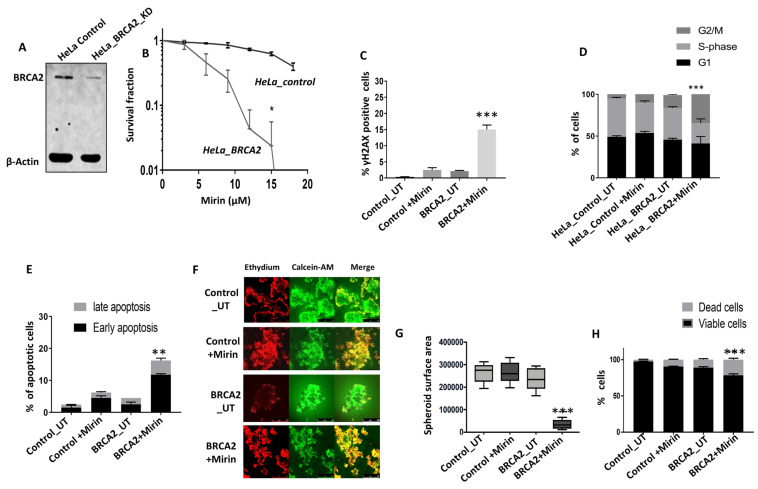
MRE11 blockade induces synthetic lethality in BRCA2-deficient HeLa cancer cells. (**A**) Western blot for BRCA2 levels in HeLa control and HeLa BRCA2_KD cells. (**B**) Mirin sensitivity in Hela control and HeLa BRCA2_KD cells (clonogenic assay). (**C**) Quantification of γH2AX-positive cells via flow cytometry in untreated and Mirin-treated HeLa control and HeLa BRCA2_KD cells (UT = untreated). (**D**) Cell cycle analysis via flow cytometry in untreated and Mirin-treated HeLa control and HeLa BRCA2_KD cells (UT = untreated). (**E**) AnnexinV analysis by flow cytometry in untreated (UT) and Mirin-treated HeLa control and HeLa BRCA2_KD cells. (**F**) Representative photomicrographic images of HeLa control and HeLa BRCA2 3D spheroids treated with Mirin (18 µM) for 48 h (UT = untreated) (magnification, ×20). (**G**) Quantification of spheroid sizes using ImageJ software (UT = untreated). (**H**) Quantification of spheroid cell viability via flow cytomery (UT = untreated). ‘*’—*p* ≤ 0.05; ‘**’—*p* ≤ 0.01; ‘***’—*p* ≤ 0.001. Figures are representative of 3 or more experiments. Error bars represent standard errors of mean between experiments.

To validate our findings in another cancer cell line model, we tested Mirin sensitivity in HeLa control and BRCA2-deficient HeLa cells (Figure 2A). As shown in Figure 2B, the HeLa_BRCA2_KD cells were extremely sensitive to Mirin treatment compared to the HeLa control cells. Increased sensitivity was associated with DSB accumulation (Figure 2C), G2/M cell cycle arrest (Figure 2D), and increased apoptosis (Figure 2E). Mirin treatment significantly reduced the HeLa_BRCA2_KD spheroid size (Figure 2F,G) and increased dead cells (Figure 2H) compared to the Mirin-treated HeLa_control spheroids.

### 2.2. In Silico Docking of Mirin onto MRE11

**Figure 3:**
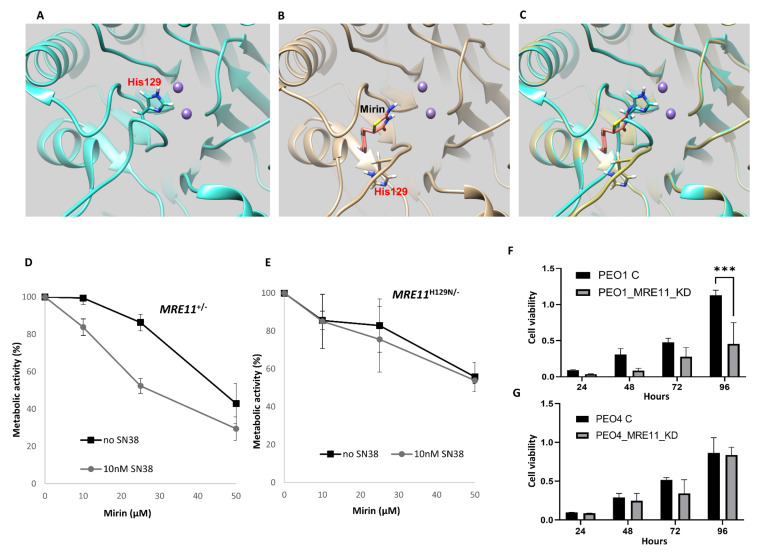
Investigating the specificity of Mirin in silico and in vitro. (**A**) In silico docking of Mirin onto MRE11. Location of His129 in the Apo structure of *H. sapiens* Mre11 (PDB: 3T1i), close to the Mn^2+^ ions (medium purple) in the active site. (**B**) Remodelling loop 127-134 of *H. sapiens* Mre11 [17] creates a conformation capable of accommodating Mirin (carbon atoms coloured salmon) that mirrors the known Mirin binding conformation of *Thermotoga maritima* MRE11 [22]. Mirin binding is associated with His129 projecting away from the active site (**C**) Overlay of panels (**A**,**B**). (**D**) Mirin sensitivity in DT40 *MRE11^+/^*^−^ cell line tested in the absence and presence of SN-38, as determined using an XTT assay. (**E**) Mirin sensitivity in nuclease-dead *MRE11^H129N/−^* cell lines tested in the absence and presence of SN-38, as determined via an XTT assay. (**F**) Cell viability in control PEO1 cells and MRE11_KD PEO1 cells, determined via an MTS assay. (**G**) Cell viability in control PEO4 cells and MRE11_KD PEO4 cells, determined via an MTS assay. ‘***’—*p* ≤ 0.001. Error bars represent standard deviations, *n* = 3.

To understand the potential mechanism of action of Mirin on MRE11, we conducted in silico docking studies. *H. sapiens* Apo MRE11 (PDB 3T1i) [29] was prepared for docking with Mirin. As shown in Figure 3A, His129, a critical residue for MRE11 nuclease activity, is located close to the Mn^2+^ ions in the active site. The Mirin binding site was identified via the co-crystallisation of Mirin with *Thermotoga maritima.* MRE11 [30] is not accessible in the known crystal structure of *H. sapiens* Apo MRE11. Remodelling loop 127–134 of *H. sapiens* MRE11 [25] creates a conformation capable of accommodating Mirin (carbon atoms coloured salmon) that mirrors the Mirin binding site in *T. maritima* MRE11. Out of a range of docked poses, Mirin has the highest-scoring binding poses at this site. Mirin binding is associated with His129 projecting away from the active site (Figure 3B,C).

### 2.3. Mirin Is Cytotoxic in DT40 MRE11^+/−^ but Not in Nuclease-Dead MRE11^H129N/−^ Cells

The in silico data presented above suggest that Mirin may dock onto the active site of MRE11 and close to the H129 amino acid residue. Previous studies have shown that H129 is a residue essential for MRE11 nuclease activity [31,32]. To provide evidence that Mirin preferentially targets MRE11 at the doses used in the current study, we tested Mirin activity in DT40 cells with functional or nuclease-dead MRE11 cells. We treated DT40 *MRE11^+/−^* and nuclease-dead *MRE11^H129N/−^* cell lines with increasing doses of Mirin in the absence or presence of the Topoisomerase I poison SN-38, the active metabolite of Irinotecan. As shown in Figure 3D,E, Mirin sensitises *MRE11^+/−^* cells to SN-38 but fails to sensitise *MRE11^H129N/−^* cells. The data confirm that the sensitising effect of Mirin is due to the specific inhibition of Mre11 nuclease activity at the doses used in the current study.

### 2.4. MRE11 Depletion Reduces Cell Viability in BRCA2-Deficient Cells

To validate a synthetic lethal interaction, we depleted MRE11 in BRCA2-deficient (PEO1) or proficient cells (PEO4) (Supplementary Figure S1A). As shown Figure 3F, cell viability was significantly reduced in the MRE11_KD PEO1 cells compared to the controls. On the other hand, MRE11_KD did not influence viability in the PEO4 cells (Figure 3G).

Taken together, the data provide evidence that MRE11 blockade or depletion is associated with selective toxicity in BRCA2-deficient ovarian cancer cells. We then proceeded to evaluate potential mechanisms of resistance to MRE11 blockade in BRCA2-deficient ovarian cancer cells.

### 2.5. Mechanism of Resistance to MRE11 Blockade in BRCA2-Deficient Cells

**Figure 4:**
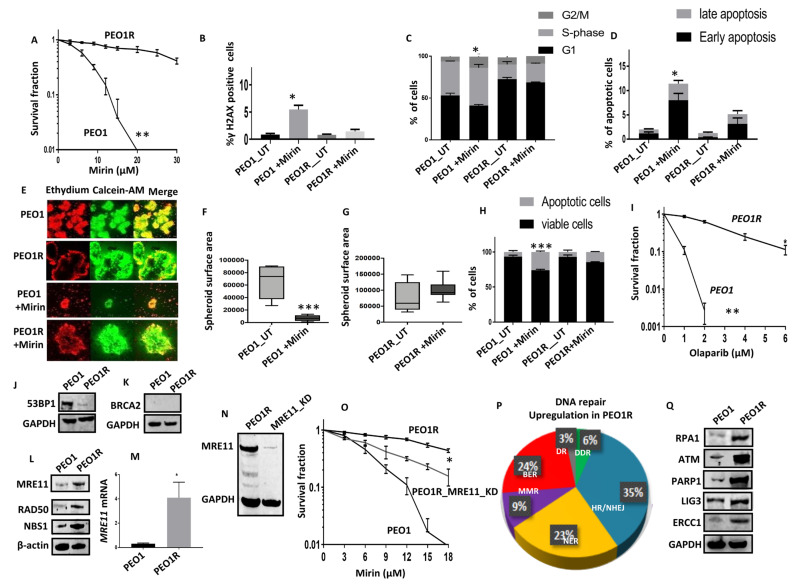
Development and evaluation of Mirin-resistant ovarian cancer cell line. (**A**) Mirin sensitivity, determined via clonogenic survival assay, in PEO1 and PEO1R cells. (**B**) γH2AX analysis via flow cytometry in Mirin-treated PEO1 and PEO1R cells (UT = untreated). (**C**) Cell cycle analysis via flow cytometry in Mirin-treated PEO1 and PEO1R cells (UT = untreated). (**D**) AnnexinV analysis via flow cytometry in Mirin-treated PEO1 and PEO1R cells (UT = untreated). (**E**) Representative photomicrographic images for PEO1 and PEO1R 3D spheroids treated with Mirin (18 µM) for 48 h (magnification, ×20). (**F**) Quantification of spheroid size using ImageJ software for PEO1 spheroids treated with Mirin or Cisplatin (UT = untreated). (**G**) Quantification of spheroid size using ImageJ software for PEO1R spheroids treated with Mirin (UT = untreated). (**H**) Quantification of spheroid cell viability via flow cytometry for PEO1/PEO1R spheroids treated with Mirin (UT = untreated). (**I**) Olaparib sensitivity, determined via clonogenic survival assay, in PEO1 and PEO1R cells. (**J**) Western blot for 53BP1 in PEO1 and PEO1R cells. (**K**) Western blot for BRCA2 in PEO1 and PEO1R cells. (**L**) Western blot for MRE11, RAD50, and NBS1 protein levels in PEO1 and PEO1R cells. (**M**) *MRE11* mRNA expression in PEO1 and PEO1R cells. (**N**) MRE11 knockdown using siRNA in PEO1R cells. (**O**) Mirin sensitivity, determined via clonogenic survival assay, in PEO1, PEO1R, and PEO1R_MRE11_KD. (**P**) Pie chart showing the upregulation of DNA repair genes that are involved in several DNA repair pathways. (**Q**) Western blot for RPA1, ATM, PARP1, LIG3, and ERCC1 protein levels in PEO1 and PEO1R cells. ‘*’—*p* ≤ 0.05; ‘**’—*p* ≤ 0.01; ‘***’—*p* ≤ 0.001. All figures are representative of 3 or more experiments. Error bars represent standard errors of mean between experiments.

PEO1 cells were treated with increasing doses of Mirin (5–45 μM) over a period of seven months. At each dose level, the PEO1 cells were maintained for three generations. The emergence of Mirin-resistant clones (PEO1R cells) was evident after seven months (Figure 4A). The enhanced repair capacity of the PEO1R cells was supported by the lack of significant DSB accumulation (Figure 4B), cell cycle alteration (Figure 4C), or apoptosis (Figure 4D) following Mirin treatment compared to the PEO1 cells. Moreover, the PEO1R spheroids were also resistant to Mirin (Figure 4E–H). In addition, the PEO1R cells were cross-resistant to the PARP inhibitor Olaparib (Figure 4I). Taken together, the data suggest that the Mirin-resistant PEO1R cells may have an increased DNA repair capacity compared to the PEO1 cells. We proceeded to further mechanical investigations.

In the PEO1R cells, we observed reductions in 53BP1 levels (Figure S1B and Figure 4J), but no obvious re-expression of BRCA2 was seen (Figure 4K) compared to the parenteral PEO1 cells. MRE11 protein overexpression was, however, evident (Figure S1C and Figure 4L), in addition to the overexpression of RAD50 (Figure S1D and Figure 4L) and NBS1 (Figure S1E and Figure 4L). We also observed an upregulation of *MRE11* transcripts (Figure 4M) in the PEO1R cells compared to the PEO1 cells. When MRE11 was depleted by siRNAs (Figure 4N), we observed partial re-sensitization to Mirin (Figure 4O) treatment in the PEO1R_MRE11_KD_ cells compared to the PEO1R cells. The parental PEO1 cells were extremely sensitive to Mirin (Figure 1A and Figure 4O). The data would suggest that the upregulation of additional DNA repair genes aside from MRE11 may also contribute to resistance observed in the PEO1R cells. To address this possibility, we performed a real-time PCR using the RT^2^ Profiler PCR Array to evaluate the expression of 84 genes involved in DNA damage signalling and repair in PEO1 and PEO1R cells (Supplementary Figure S1F–H, Supplementary Table S1). We not only confirmed *MRE11 mRNA* overexpression in PEO1R cells but also observed the overexpression of a number of genes involved in DNA repair, including DDR signalling (*ATM*, *RPA1*), HR (*RAD51B*, *RAD51C*, *RAD51D*, *RAD52*, *TOP3A*, *TOP3B*, *LIG1*, *LIG3)* NHEJ (*XRCC5*, *XRCC6BP1*, *LIG4*,), nucleotide excision repair (*RAD23B*, *DDB1*, *DMC1*, *XPA*, *ERCC1*, *ERCC2*, *ERCC4*, *ERCC8* and *XAB2*), mismatch repair (*MSH3*, *MSH5*, *PMS2*), base excision repair (*APEX1*, *TDG*, *NEIL1*, *NEIL2*, *MPG*, *MUTYH*, *PARP1*, *Polβ*, *LIG1*, *LIG3*), and MGMT (Figure S1G,H and Figure 4P). Using a panel of antibodies, we confirmed the overexpression of ATM, PARP1, LIG3, ERCC1, and RPA1 protein in the PEO1R cells compared to the PE01 cells (Figure S2 and Figure 4Q). Together, the data suggest that the upregulation of multiple DNA repair pathway genes, including MRE11, in PEO1R cells compared to PEO1 cells contributes to Mirin resistance.

#### In Vivo Xenograft Studies

**Figure 5:**
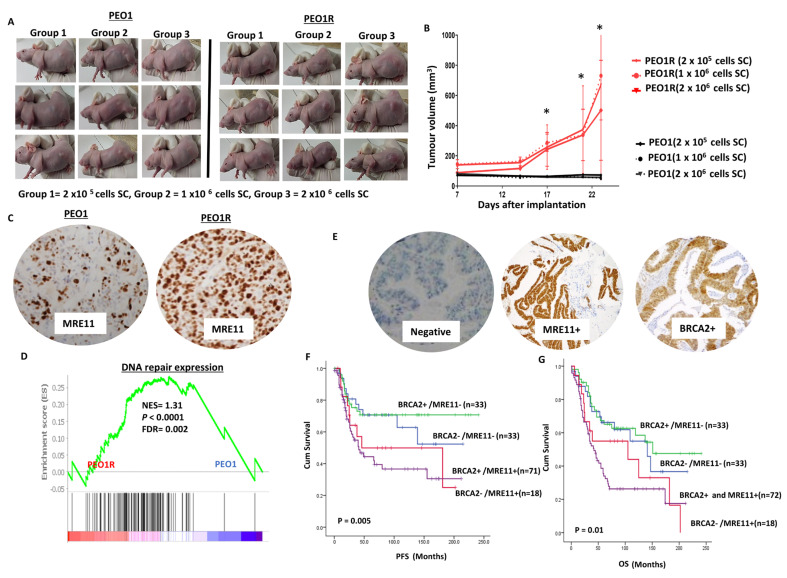
(**A**) The tumour volume in PEO1-bearing CD-1 NuNu mice compared to PEO1R-bearing CD-1 NuNu mice (see methods for details; 3 mice were used per group). (**B**) Tumour volumes in PEO1R were substantially larger in PEO1R-bearing CD-1 NuNu mice compared to PEO1-bearing CD-1 NuNu mice (‘*’—*p* ≤ 0.05). (**C**) Immunohistochemical expressions of MRE11 in PEO1 and PEO1R xenografts (magnification, ×20). (**D**) GSEA comparing PEO1R vs. PEO1 signatures to the DNA repair gene set. The graphical representation shows significant enrichments in the DNA repair genes (shown as black lines) in PEO1R cells (red bar) compared to PEO1 cells (represented as the blue bars). Normalized enrichment scores (NES), nominal *p*-values, and FDR corrected *p*-values shown. GSEA comparing PEO1R vs. PEO1 signatures to the DNA repair gene set rare shown here. (see Section 2 for more details). (**E**) Representative photomicrographic images of negative, MRE11 overexpression, and BRCA2 overexpression in human ovarian tumours (magnification, ×20). (**F**) BRCA2-MRE11 co-expression and Kaplan–Meier curves for progression-free survival (PFS) in ovarian cancer. The *p*-values indicate univariate overall comparisons between BRCA2+/MRE11+, BRCA2+/MRE11−, BRCA2−/MRE11+, and BRCA2−/MRE11+ tumours. (**G**) MRE11and BRCA2 co-expression and Kaplan–Meier curves for overall survival in ovarian cancer.

We then conducted in vivo tumorigenicity studies. As shown in Figure 5A,B, the tumour volumes were substantially higher in PEO1R-bearing CD-1 NuNu immunodeficient mice compared to PEO1-bearing CD-1 NuNu immunodeficient mice. An IHC evaluation confirmed MRE11 overexpression in the PEO1R xenografts (mean H-score = 250) compared to the PEO1 xenografts (mean H-score = 50) (Figure 5C). RNA sequencing and a gene set enrichment analysis (GSEA) revealed significantly enriched DNA repair gene expression signature profiles in the PEO1R xenografts compared to the PEO1 xenografts (Figure 5D). The data provide in vivo evidence of DNA repair upregulation, including MRE11, in the PEO1R xenografts compared to the PEO1 xenografts. As the PEO1R xenografts were highly tumorigenic, we speculated that MRE11 could also have predictive and/or prognostic significance in human tumours. We therefore proceeded to an immunohistochemical evaluation of MRE11-BRCA2 co-expression in human ovarian cancer.

### 2.6. MRE11-BRCA2 Co-Expression in Human Ovarian Cancers

We have recently shown that MRE11 overexpression is a predictive biomarker of platinum resistance in human ovarian cancer tumours [25]. Here, we conducted immunohistochemical studies of BRCA2 and MRE11 co-expression in 226 evaluable sporadic epithelial ovarian cancers. Patient demographics are summarised in Supplementary Table S3. Patients whose tumours had low levels of expression of BRCA2 with high levels of MRE11 co-expression (Figure 5E) had worse PFS (*p* = 0.005, Figure 5F) and overall survival (*p* = 0.001, Figure 5G) compared to patients whose tumours had high levels of BRCA2 with low levels of MRE11 co-expression. The clinical data confirm that in BRCA2-deficient sporadic ovarian cancers, MRE11 blockade could be a viable strategy.

The cBioPortal [33] was utilised to analyse the ovarian serous cystadenocarcinoma TCGA dataset (TCGA, Firehose Legacy—311 samples/patients). *MRE11* was not mutated in any of the samples, and 7% of samples (21/311 samples) had copy number alterations (3 samples had deep deletions and 19 samples were amplified). *BRCA2* was mutated in 12 samples, with the mutations leading to shallow deletions (frameshift deletions and nonsense mutations). *BRCA2* copy number variations were seen in 11 samples (3 samples with deep deletions and 8 samples with amplification). Interestingly, there was no significant co-occurrence of the genetic alterations (FDR corrected *p*-value, q-value = 0.474). We observed a weak correlation of the mRNA levels (182 samples, Spearman 0.16, *p* = 0.0265; Pearson correlation 0.12, *p* = 0.115) (Figure S3).

## 3. Discussion

**Figure 6:**
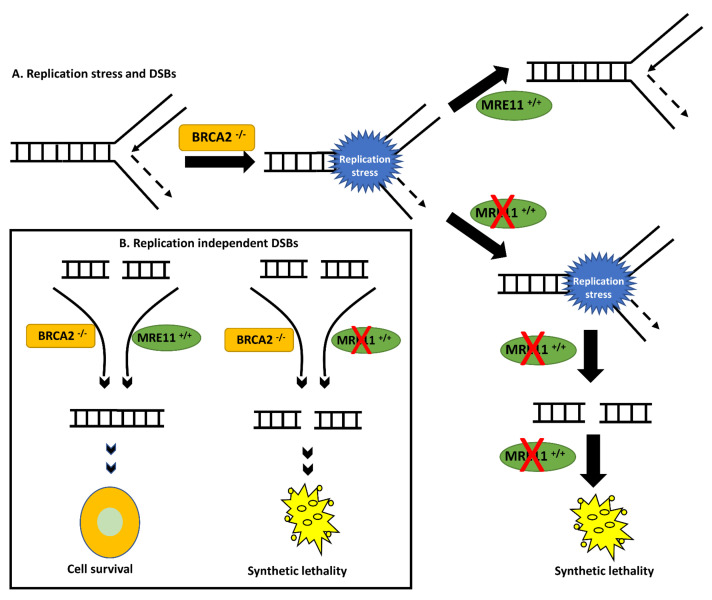
A model for MRE11-blockade-induced synthetic lethality. (**A**). BRCA2-deficiency results in replication stress. MRE11 blockade prevents resolution of replication stress, leading to DSB accumulation and cell death (**B**). Replication-independent DSBs can be generated endogenously (e.g., free-radical-induced DNA damage) in BRCA2-deficient cells. MRE11 blockade can lead to DSB accumulation, cell cycle arrest, and cell death. DSB = double-strand breaks.

MRE11 is critical for genomic stability [18,19,20]. Here, we show that MRE11 blockade by the small molecule inhibitor Mirin is synthetically lethal in BRCA2-deficient cells. A model is proposed as follows (Figure 6): Firstly, when DSBs are generated endogenously (e.g., free radicals), BRCA2-deficient cells will be reliant on MRE11-dependent alternative DSB repair pathways for survival [34,35]. Given the role of RAD52 in the maintenance of cellular survival in BRCA2-deficient cells [10,11,12,13,14], we speculate that MRE11 may channel DSB repair through RAD52-mediated error-prone pathways. Moreover, an MRE11 blockade will prevent ATM activation, which may block compensatory DSB repair, leading onto DSB accumulation, cell cycle arrest, and cell death [36]. Secondly, MRE11 plays an important role during the restart of stalled replication forks [18,19,20]. BRCA2 also protects stalled replication forks from degradation by the MRE11 nuclease [26]. In BRCA2-deficient cells, MRE11 is hyper-activated to promote survival. An MRE11 blockade in BRCA2-deficient cells results in replication stress, which leads to DSB accumulation and cell death (Figure 6). Accordingly, in BRCA2-deficient cells we observed significant sensitivity to Mirin treatment, which was associated with DSB accumulation, G2/M cell cycle arrest, and increased apoptosis. In clinical cohorts, we observed that patients with low levels of BRC2 expression and high-MRE11 ovarian tumours were associated with worse PFS and OS outcomes.

Whilst we cannot completely exclude that Mirin may have blockade activity beyond MRE11, for the doses tested in this study, we provide evidence that Mirin has selective activity against MRE11. Firstly, in silico modelling suggests that Mirin docks avidly onto the active site close to H129 and Mn^2+^. We also observed that Mirin binds a conformation in which H129 is projecting away from the active site, which may explain its mechanism of action. Secondly, in nuclease-dead DT40 cells, Mirin was unable to increase cytotoxicity of SN-38 a topoisomerase 1 poison in contrast to DT40 cells with functional MRE11.

The lessons learnt from the development of resistance to PARP inhibitors suggest that the restoration of HR (reactivation of BRCA function and inactivation of 53BP) and the restoration of stalled replication fork protection are key routes to resistance [37]. Here, in Mirin-resistant cells, we observed 53BP1 inactivation and the upregulation of multiple DNA repair genes, including MRE11. In xenograft studies, we also confirmed DNA repair upregulation as a key route to resistance. Although the data provide novel insights into the development of resistance to the MRE11 inhibitor in BRCA2-deficient cells, further mechanistic studies will be required to understand the mechanism of DNA repair upregulation in Mirin-resistant cells.

In a clinical cohort of ovarian cancer, we show that the patients whose tumours have high levels of expression of MRE11 and low levels of expression of BRCA2 have poor rates of PFS, implying that targeting MRE11 could be clinically relevant. In conclusion, we provide evidence that MRE11 is an attractive target. The pharmaceutical development of MRE11 inhibitors is likely to have clinical impact.

## 4. Materials and Methods

### 4.1. Pre-Clinical Study

#### 4.1.1. Cell Lines and Tissue Culture

PE01 (BRCA2 deficient) and PE04 (BRCA2 proficient) cells were purchased from American Type Culture Collection (ATCC, Manassas, VA, USA). The cells were cultured in RPMI (R8758, Merck, Feltham, UK) and supplemented with 10% FBS (F4135, Merck, UK) and 1% penicillin–streptomycin (P4333, Merck, UK). HeLa control and BRCA2 HeLa SilenciX cells were purchased from Tebu-Bio, Peterborough, UK). The HeLa control cells were cultured in Dulbecco’s Modified Eagle Medium (D6429, Sigma, Welwyn Garden City, UK), supplemented with 10% FBS and 1% penicillin streptomycin, while the BRCA2 HeLa SilenciX cells were grown in Dulbecco’s Modified Eagle’s Medium-high glucose (D6429) and supplemented with 10% FBS, 1% penicillin/streptomycin, and 125 μg/mL of hygromycin B (H0654, Sigma, UK). All cell lines were maintained in a humidified incubator at 37 °C in a 5% CO_2_ atmosphere.

DT40 *MRE11^+/−^* and nuclease-dead *MRE11^H129N/−^* cells were created and cultured as previously described [38]. Briefly, DT40 cells were cultured in RPMI 1640 media with 10% FBS, 1% chicken serum, and 1% penicillin/streptomycin. The DT40 *MRE11^H129N/^^−^* nuclease mutant was created from *MRE11^+/−^* cells via a targeted knock-in, leaving *MRE11* under the control of its own promoter and apart from the H129N mutation in exon 4 and the integration of a puromycin resistance marker in the intron downstream of this mutation, leaving the overall intron/exon structure intact. For the XTT assay in 96-well plates, DT40 cells (50,000 per well) were incubated for 4 h at 39 °C, after which drugs and compounds were applied. The cells were incubated for an additional 48 h at 39 °C, XTT reagent (20 μL) was added to each well, and the cells were incubated for 4 h. Absorbances were read at 450 nm using a microplate reader and corrected for the absorbances of wells containing medium without cells or drugs. Rates of metabolic activity were expressed as a percentage relative to the untreated control cells. Metabolic activity was measured using an XTT assay [39].

#### 4.1.2. Western Blot Analysis

The cells were harvested and lysed in RIPA buffer (R0278, Sigma, UK) with the addition of a protease cocktail inhibitor (P8348, Sigma, UK), phosphatase inhibitor cocktail 2 (P5726, Sigma, UK), and phosphatase inhibitor cocktail 3 (P0044, Sigma) and stored at −20 °C. Proteins were quantified using a BCA Protein Assay kit (23225, Thermofisher, Horsham, UK). Samples were run on an SDS bolt bis-tris gel (4–12%). Th membranes were incubated with primary antibodies as follows: MRE11 (1:500, ab214), RAD50 (1:500, ab89), NBS1 (1:500, N3162-200UL, Siqma, UK), ERCC1 (1:2000, M3648, DAKO, Sanat Clara, CA, USA), ß-actin (1:1000, ab8226), YY1 (1:1000, ab109228), GADPH (1:1000, ab9485), BRCA2 (1:2000, ab123491), N-Cad (1:1000, ab18203), E-cad (1:1000, 4A2C7, Thermofisher, Horsham, UK), ATM antibody (1:1000, ab32420), OCT4 (1:1000, 27505, Cell Signalling, Leiden, Netherlands), LIG3 (1:2000, Sigm, HPA006723, 100UL), C-MYC (1:500, ab32), ALDH1A1 (1:1000, D4R9V, Cell Signalling) and SOX2 (1:1000, 2748S, Cell Signalling), CD44 (1:1000, ab51037), 53BPI (1:1000, Cell Signalling, 4937S), PARP1 (1:2000, ab32138), and RPA1 (1:1000, ab79398, Abcam, Cambridge, UK). The membranes then were washed and incubated with infrared-dye-labelled secondary antibodies (LiCor) (IRDye 800CW Donkey Anti-Rabbit IgG (926-32213) and IRDye 680CW Donkey Anti-Mouse IgG (926-68072)) at a dilution of 1:10,000 for 1 h. The membranes were scanned using a LiCor Odyssey machine (700 and 800 nm) to determine their protein levels.

#### 4.1.3. Transient Knockdowns of MRE11

MRE11 (ID S8960) and the validation construct (MRE11 (S S8960)) siRNAs’ oligonucleotides were obtained from Invitrogen. A Lipofectamine 3000 reagent (L3000015, Invitrogen, Altrincham, UK) was used according to the manufacturer’s protocol. Briefly, the cells were seeded at 50–60% confluency in T25 flasks overnight. The cells were then transfected with 20 nM of siRNA oligonuclotide or scrambled SiRNA oligonucleotide control (4390843, Thermofisher) in Opti-MEM media (31985-062, Gibco, Fisher Scientific, Loughborugh, UK). Transfection efficiency was confirmed via Western blot.

#### 4.1.4. MTS Assay

For the MTS assay, 1000 cells/per well were seeded onto 96-well plates. The plates were incubated at 37 °C and 5% CO_2_. After 24 h, 48 h, 72 h, and 96 h, the MTS reagent (CellTiter 96^®^ AQueous One Solution, Promega, Southampton, UK) was added, and the plates were incubated in dark conditions for 4 h at 37 °C. The FlUOstar Optima plate reader was employed at 490 nm to evaluate formazan absorbance.

#### 4.1.5. Clonogenic Assays

In the clonogenic assay, 32 cells/cm^2^ were seeded in 6-well plates and left at 37 °C in a 5% CO_2_ atmosphere. Cisplatin (kindly provided by Nottingham University Hospital) or Mirin (M9948, Sigma, UK) were added at the indicated concentrations, and the plates were left at 37 °C in a 5% CO_2_ atmosphere for 14 days. Later the plates were washed with PBS, fixed and stained, and colonies were counted.

#### 4.1.6. Functional Studies

First, 1 × 10^5^ cells per well were seeded in 6-well plates and left overnight at 37 °C in a 5% CO_2_ atmosphere. After 24 h, 5 µM of Cisplatin or 18 µM or 25 µM of Mirin were added to cells and incubated for 24 h and 48 h. The cells then were collected via trypsinisation, washed with ice-cold PBS, and fixed in 70% ethanol for 1 h at −20 °C. After the removal of the fixative solution via centrifugation, for the DNA double-strand break analysis, the cells were stained with 2 mg/mL of phospho-Histone (γH2AX) Ser139 (16202A, Millipore, Watford, UK). For the cell cycle analysis, the cells were treated with 20 mg/mL of RNase A (12091021, Invitrogen), and then 10 mg/mL of Propidium Iodide (P4170, Sigma Aldrich, Gillingham, UK) was added to determine the cell cycle distribution. The samples were analysed on a Beckman-Coulter FC500 flow cytometer using a 488 nm laser for excitation and emission data for PI collected using a 620 nm bandpass filter (FL3) and a 525 nm bandpass filter (FL1) for FITC-anti-phospho-Histone H2A.X. For the apoptosis assay, the cells were analysed using an Annexin V detection kit (556547, BD Biosciences, Plymouth, UK). Briefly, the cells were trypsinised, washed with PBS, and the cellular pellet was re-suspended in Annexin Binding Buffer (1×). Then, 2.5 mL of FITC Annexin V and 2.5 mL of Propidium Iodide were added to the cells. After incubation, 300 mL of Annexin Binding Buffer (1×) was added to each tube. The samples were analysed on a Beckman-Coulter FC500 flow cytometer. The data were analysed using Weasel software (version 3.0.2). Graphical representations and a statistical analysis were carried out using GraphPad Prism 7 (GraphPad, La Jolla, CA, USA).

#### 4.1.7. Generation of 3D Spheroids

First, 4 × 10^4^ cells per well were plated in ultra-low-attachment 6-well plates in a Promocell serum-free tumour spheres medium (C-28070). The cells were then topped off with fresh medium every three days until spheroid structures were formed. The spheroids were treated with cisplatin or a Mirin inhibitor for 48 h. To quantify cell viability, a LIVE/DEAD Viability/Cytotoxicity Kit (L3224, Thermo Fisher Scientific) was used. Briefly, the spheroids were collected via trypsinisation, washed with PBS, and centrifuged at 1000× *g* for 5 min. The light-protected cellular pellet in PBS was loaded with 0.1 µM of Calcein-AM and 1 µM of Ethidium homodimer-1 for 20 min at room temperature. The samples were then analysed on a Beckman-Coulter FC500 flow cytometer using a 495 nm laser for excitation and a 515 nm laser for emission data for Calcein AM and a 495 nm laser for excitation and emission at 635 nm for Ethidium Homodimer-1. In addition, Image J software was used to calculate spheroid diameters. The mean of three diagonal diameters was taken as diameter for each spheroid. At least 10 spheroids were measured.

#### 4.1.8. Immunofluorescence Staining

The cells were seeded on cover slips overnight, fixed with 4% paraformaldehyde (8187085000, Sigma) for 30 min, washed with PBS, and permeabilized with 0.1% triton (HFH10, Thermofisher) for 30 min. The cells were blocked with 3% BSA (A7906, Sigma) for 1 h. The cells were incubated with OCT 4 (1:50) and MRE11 (1:100) overnight at 4 °C. The slides were then washed and incubated with goat anti-rabbit (A16129, Invitogen, UK) and goat anti-mouse (A11029, Invitrogen, UK) for 1 h. Imaging was carried out using a Leica confocal microscope. For analysis, at least 100 cells per slides were counted.

#### 4.1.9. Real-Time PCR

For the DNA-repair-pathway-focused analysis, RT2 PCR array plates were used (PAHS-042ZC, Qiagen, Manchester, UK) (Supplementary Table S1). RNA was extracted using an RNeasy Mini kit (74104, Qiagen), and cDNA conversion carried out using an RT2 first strand kit (330404, Qiagen, Manchester, UK), as per the manufacturer’s protocol. Samples were run on ABI-7500 fast block. The data were analysed as per the manufacturer’s recommendations. A real-time PCR was carried out on an Applied Biosystems 75000 FAST cycler.

#### 4.1.10. Development of Mirin-Resistant PEO1R Cell Line

The PEO1 cells were treated with increasing doses of Mirin treatment (5–45 μM). A starting dose of 5 μM of was chosen (based on IC50 of Mirin in the PEO1 cells). At each dose level, the PEO1 cells were maintained for three generations. Mirin-resistant PEO1R cells were established over a period of seven months.

#### 4.1.11. Tumour Xenograft Studies

In vivo tumorgenicity experiments were performed to establish the growth rates of the two cell lines, PEO1 and PEO1R, at different cell concentrations. The experiments were conducted under the UK Home Office Project Licence number PPL P435A9CF8. LASA good practice guidelines, and the FELASA working group on pain and distress guidelines and the ARRIVE reporting guidelines were also followed. Eighteen female immunodeficient CD-1 NuNu mice at 7–8 weeks old were purchased from Charles River UK. The mice were maintained in individually ventilated cages (Tecniplast UK, Northampton, UK) within a barriered unit, illuminated by fluorescent lights set to provide a 12 h light–dark cycle (on 07.00, off 19.00), as recommended in the guidelines from the Home Office Animals (Scientific Procedures) Act 1986 (UK). The room was air-conditioned by a system designed to maintain an air temperature range of 21 ± 2 °C and a humidity of 55% ± 10%. Durin the study, the mice were housed in social groups, three per cage, with irradiated bedding and autoclaved nesting materials and environmental enrichment (Datesand UK, Stockport, UK). A sterile, irradiated 5V5R rodent diet (IPS Ltd., London, UK) and irradiated water were offered ad libitum. The animals’ conditions were monitored throughout the study by an experienced animal technician. After a week’s acclimatisation, the mice were initiated with tumours as follows.

The cells were maintained in vitro in RPMI culture medium (Sigma, UK) containing 10% (*v*/*v*) heat-inactivated foetal bovine serum (Sigma, Poole, UK) and 2 mM of L-glutamine (Sigma, UK) at 37 °C in 5% CO_2_ and humidified conditions. Cells from sub-confluent monolayers were harvested with 0.025% EDTA, washed in culture medium, and counted. Cells with a viability of >90% were re-suspended and seeded at 2 × 10^6^ cells per T150 flask and incubated for 48 h. On the day of initiation, cells were harvested from semi-confluent monolayers with 0.025% EDTA, washed twice in the culture medium, and counted three times, as above. Cells with viability of >90% were re-suspended for in vivo administration in standard matrigel at 2 × 10^5^, 1 × 10^6^, and 2 × 10^6^/100 µL for subcutaneous injection into the left flank. Three mice were implanted per cell concentration for each of the two cell lines. Tumour establishment and growth were measured using Vernier callipers (Camlab, Cambridge, UK) twice weekly, and the animals were weighed weekly. The mice were also photographed weekly to record tumour development. The scientific end point was determined to be when the first tumour reached the maximum allowable size under the PPL, i.e., 1.2 cm in diameter. At this point, all mice were culled via cervical dislocation, and the tumours were dissected out and photographed ex vivo. The tumours were preserved via snap-freezing, fixation (NBF) 50:50, and by freezing in RNA later.

#### 4.1.12. RNA Seq Analyses

RNA quality and integrity (RIN > 9.9) were confirmed using a Nanodrop spectrophotometer and an Agilent 2100 Bioanalyzer, respectively. Library preparation and Illumina sequencing were completed (Novogene Co., Ltd., Cambridge, UK). Raw data were been deposited into the NCBI-GEO in fastq format with the following accession numbers: GSE198648 and GSE160540. Paired-end RNA sequencing data in fastq format were assessed for quality using the Trimgalore wrapper for FastQC and Cutadapt (http://www.bioinformatics.babraham.ac.uk/projects/trim_galore/). Contaminating adapter sequences were removed, and high-quality reads with phred scores <30 were retained. The resultant fastq files were aligned to the Ensembl annotated human reference genome (GRCh38) using the STAR aligner [40], and gene expression was quantified using FeatureCounts [41]. The pathway analysis was completed using the GSEA platform [42].

#### 4.1.13. Gene Set Enrichment Analysis

We used a gene set enrichment analysis (GSEA) (www.broadinstitute.org/gsea, [42]) to assess the degrees of association between our signature and other signatures previously identified. A GSEA requires ranking genes (as PEO1R vs. PEO1) to determine whether genes in a signature tend to present either high (positively enriched genes) or low ranks (negatively enriched genes). The outputs of GSEA are an enrichment score (ES) and a normalized enrichment score (NES), which accounts for the size of the gene set being tested and a *p* value. The nominal *p*-value estimates the statistical significance of the enrichment score for a single gene set. ESs, NESs, and *p*-values were obtained as previously proposed [42]. We derived an independent DNA repair expression signature [43] by selecting those genes with predominant roles in DNA repair (Supplementary Table S2).

#### 4.1.14. Statistical Analysis

A statistical analysis was conducted as on GraphPad Prism7 software (GraphPad, La Jolla, CA, USA). To compare between two groups, Student’s *t*-test was performed. A one-way ANOVA was performed to compare between more than two groups (variance analyses). A two-way ANOVA was used to analyse two variables, such as the results of the Annexin V analysis and cell cycle analysis. All experiments were expressed as means ± standard deviations, S.D.s, of three independent experiments. *p*-values < 0.05 = *, *p*-value < 0.01 = **, and *p*-value < 0.001 = ***.

#### 4.1.15. In Silico Mirin Docking Studies

Molecular graphics and analyses were performed using UCSF Chimera, which was developed by the Resource for Biocomputing, Visualization, and Informatics at the University of California, San Francisco, with support from NIH P41-GM103311 [44]. *H. sapiens* Apo MRE11 (PDB 3T1I) was prepared for docking using Dockprep under default settings. Loop 127–134 was remodelled employing Modeller [45] to define a human MRE11 template as published [25]. This template was then used as the receptor file to dock Mirin using Autodock Vina [46,47]. The top-scoring binding pose was taken.

### 4.2. Clinical Study

#### 4.2.1. MRE11 Expression Levels in Ovarian Cancers

An investigation of the expression levels of MRE11 and BRCA2 was carried out on tissue microarrays of 331 consecutive ovarian epithelial cancer cases treated at Nottingham University Hospitals (NUH) between 1997 and 2010. This study was carried out in accordance with The Declaration of Helsinki and ethical approval, which was obtained from the Nottingham Research Ethics Committee (REC Approval Number 06/Q240/153). The characteristics of this cohort are summarized in Supplementary Table S3.

#### 4.2.2. Tissue Microarray (TMA) and Immunohistochemistry (IHC)

Tumours were arrayed in tissue microarrays (TMAs) constructed with two replicate 0.6 mm cores from the tumours. Immunohistochemical staining was conducted using the Thermo Fisher Scientific Shandon Sequenza chamber system (REF: 72110017) in combination with the Novolink Max Polymer Detection System (RE7280-K: 1250 tests) and the Leica Bond Primary Antibody Diluent (AR9352); each were used according to the manufacturer’s instructions (Leica Microsystems). The tissue slides were deparaffinised with xylene and then rehydrated using five decreasing concentrations of alcohol (100%, 90%, 70%, 50%, and 30%) for two minutes each. Pre-treatment antigen retrieval was carried out on the TMA sections using a sodium citrate buffer (pH 6.0) and heated for 20 min at 95 °C in a microwave (Whirlpool JT359 Jet Chef 1000 W). A set of slides was incubated with the primary anti-MRE11 mouse monoclonal antibody (clone ab214, Abcam, Cambridge, UK), at a dilution of 1:800 for 1 h at room temperature. A set of TMA slides was incubated for 15 min at room temperature with 1:200 anti-XRCC1 mouse monoclonal antibody (Ab-1, clone 33-2-5, Thermoscientific, Fremont, CA, USA). A set of TMA slides was incubated for 60 min at room temperature with 1:25 anti-OCT4 rabbit monoclonal antibody (27505, Cell Signalling, UK).

Negative (by omission of the primary antibody and IgG-matched serum) and positive controls were included in each run.

#### 4.2.3. Evaluation of Immune Staining

A whole field inspection of the core was scored, and the subcellular localisations of each marker were identified (nuclear, cytoplasm, or cell membrane). The intensities of the subcellular compartments were each assessed and grouped as follows: 0 = no staining, 1 = weak staining, 2 = moderate staining, and 3 = strong staining. The percentage of tumour cells in each category was estimated (0–100%). A histochemical score (H-score) (range 0–300) was calculated by multiplying the intensity of staining and the percentage of staining. A median H-score of ≤110 and ≤60 was used as the cut-off for high levels of MRE11 nuclear and cytoplasmic expressions, respectively. OCT4 nuclear positivity was defined as an H-score >0. OCT4 cytoplasmic positivity was defined as a median H-score > 5.

#### 4.2.4. Statistical Analysis

Associations with clinical and pathological parameters using categorised data were examined using the Chi-squared test. All tests were two-tailed. Survival rates were determined using Kaplan–Meier method and compared using the log-rank test. All analyses were conducted using the Statistical Package for the Social Sciences (SPSS, version 22, Chicago, IL, USA) software for windows. A *p*-value of less than 0.05 was identified as statistically significant.

#### 4.2.5. Genomic and Transcriptomic Analysis in the TCGA Data Set

Analyses of the *MRE11* and *BRCA2* mutations, copy number alterations, and mRNA levels of 311 TCGA-OV specimens (TCGA Firehose Legacy) were performed using CBioportal [33]. The TCGA ovarian cancer (TCGA, Nature 2011) RNAseq expression data were obtained from GDC (https://portal.gdc.cancer.gov/). The *MRE11* and *BRCA2* expression data in counts format were accessed using the GDC portal [48].

## 5. Conclusions

The *BRCA2* tumour suppressor gene is essential for homologous recombination (HR) repair and replication fork stability maintenance. MRE11 plays a critical role during the sensing, processing, and signalling of DNA double-strand breaks. MRE11 also contributes to the processing of stalled replication forks. In the current study, we have pre-clinically shown that a synthetic lethality relationship exists between BRCA2 and MRE11. Our IHC clinical data provide evidence that MRE11 overexpression in BRCA2-deficient somatic tumours can influence aggressive clinicopathological phenotypes. We conclude that MRE11 is an attractive anti-cancer target and that the pharmaceutical development of MRE11 inhibitors for precision oncology therapeutics may have clinical benefit.

## Data Availability

Data supporting the study can be found in the Supplementary Materials File, and the corresponding author can make any materials available upon request. Aggregate data from the referenced datasets are available from the corresponding author upon reasonable request.

## Supplementary Materials

The following supporting information can be downloaded at: https://www.mdpi.com/article/10.3390/ijms241310966/s1.
