# Supplementary material for: Selective Killing of BRCA2-Deficient Ovarian Cancer Cells via MRE11 Blockade
Source: Int J Mol Sci. 2023 Jun 30;24(13):10966. doi: 10.3390/ijms241310966 (PMC10341502; doi:10.3390/ijms241310966)
Supplement: Supplementary file 1 [file ijms-24-10966-s001.zip › ijms-2418451-supplementary.pdf]

Supplementary Table S1

Position	Symbol	Description
A01	APEX1	APEX nuclease (multifunctional DNA repair enzyme) 1
A02	APEX2	APEX nuclease (apurinic/apyrimidinic endonuclease) 2
A03	ATM	Ataxia telangiectasia mutated
A04	ATR	Ataxia telangiectasia and Rad3 related
A05	ATXN3	Ataxin 3
A06	BRCA1	Breast cancer 1, early onset
A07	BRCA2	Breast cancer 2, early onset
A08	BRIP1	BRCA1 interacting protein C-terminal helicase 1
A09	CCNH	Cyclin H
A10	CCNO	Cyclin O
A11	CDK7	Cyclin-dependent kinase 7
A12	DDB1	Damage-specific DNA binding protein 1, 127kDa
B01	DDB2	Damage-specific DNA binding protein 2, 48kDa
B02	DMC1	DMC1 dosage suppressor of mck1 homolog, meiosis-specific homologous recombination
B03	ERCC1	Excision repair cross-complementing rodent repair deficiency, complementation group 1
B04	ERCC2	Excision repair cross-complementing rodent repair deficiency, complementation group 2
B05	ERCC3	Excision repair cross-complementing rodent repair deficiency, complementation group 3
B06	ERCC4	Excision repair cross-complementing rodent repair deficiency, complementation group 4
B07	ERCC5	Excision repair cross-complementing rodent repair deficiency, complementation group 5
B08	ERCC6	Excision repair cross-complementing rodent repair deficiency, complementation group 6
B09	ERCC8	Excision repair cross-complementing rodent repair deficiency, complementation group 8
B10	EXO1	Exonuclease 1
B11	FEN1	Flap structure-specific endonuclease 1
B12	LIG1	Ligase I, DNA, ATP-dependent
C01	LIG3	Ligase III, DNA, ATP-dependent
C02	LIG4	Ligase IV, DNA, ATP-dependent
C03	MGMT	O-6-methylguanine-DNA methyltransferase
C04	MLH1	MutL homolog 1, colon cancer, nonpolyposis type 2 (*E. coli*)
C05	MLH3	MutL homolog 3 (*E. coli*)
C06	MMS19	MMS19 nucleotide excision repair homolog (*S. cerevisiae*)
C07	MPG	N-methylpurine-DNA glycosylase
C08	MRE11A	MRE11 meiotic recombination 11 homolog A (*S. cerevisiae*)
C09	MSH2	MutS homolog 2, colon cancer, nonpolyposis type 1 (*E. coli*)
C10	MSH3	MutS homolog 3 (*E. coli*)

C11	MSH4	MutS homolog 4 (*E. coli*)
C12	MSH5	MutS homolog 5 (*E. coli*)
D01	MSH6	MutS homolog 6 (*E. coli*)
D02	MUTYH	MutY homolog (*E. coli*)
D03	NEIL1	Nei endonuclease VIII-like 1 (*E. coli*)
D04	NEIL2	Nei endonuclease VIII-like 2 (*E. coli*)
D05	NEIL3	Nei endonuclease VIII-like 3 (*E. coli*)
D06	NTHL1	Nth endonuclease III-like 1 (*E. coli*)
D07	OOG1	8-oxoguanine DNA glycosylase
D08	PARP1	Poly (ADP-ribose) polymerase 1
D09	PARP2	Poly (ADP-ribose) polymerase 2
D10	PARP3	Poly (ADP-ribose) polymerase family, member 3
D11	PMS1	PMS1 postmeiotic segregation increased 1 (*S. cerevisiae*)
D12	PMS2	PMS2 postmeiotic segregation increased 2 (*S. cerevisiae*)
E01	PNKP	Polynucleotide kinase 3'-phosphatase
E02	POLB	Polymerase (DNA directed), beta
E03	POLD3	Polymerase (DNA-directed), delta 3, accessory subunit
E04	POLL	Polymerase (DNA directed), lambda
E05	PRKDC	Protein kinase, DNA-activated, catalytic polypeptide
E06	RAD18	RAD18 homolog (*S. cerevisiae*)
E07	RAD21	RAD21 homolog (*S. pombe*)
E08	RAD23A	RAD23 homolog A (*S. cerevisiae*)
E09	RAD23B	RAD23 homolog B (*S. cerevisiae*)
E10	RAD50	RAD50 homolog (*S. cerevisiae*)
E11	RAD51	RAD51 homolog (*S. cerevisiae*)
E12	RAD51B	RAD51 homolog B (*S. cerevisiae*)
F01	RAD51C	RAD51 homolog C (*S. cerevisiae*)
F02	RAD51D	RAD51 homolog D (*S. cerevisiae*)
F03	RAD52	RAD52 homolog (*S. cerevisiae*)
F04	RAD54L	RAD54-like (*S. cerevisiae*)
F05	RFC1	Replication factor C (activator 1) 1, 145kDa
F06	RPA1	Replication protein A1, 70kDa
F07	RPA3	Replication protein A3, 14kDa
F08	SLK	STE20-like kinase
F09	SMUG1	Single-strand-selective monofunctional uracil-DNA glycosylase 1
F10	TDG	Thymine-DNA glycosylase
F11	TOP3A	Topoisomerase (DNA) III alpha
F12	TOP3B	Topoisomerase (DNA) III beta
G01	TREX1	Three prime repair exonuclease 1
G02	UNG	Uracil-DNA glycosylase
G03	XAB2	XPA binding protein 2
G04	XPA	Xeroderma pigmentosum, complementation group A
G05	XPC	Xeroderma pigmentosum, complementation group C
G06	XRCC1	X-ray repair complementing defective repair in Chinese hamster cells 1
G07	XRCC2	X-ray repair complementing defective repair in Chinese hamster cells 2
G08	XRCC3	X-ray repair complementing defective repair in Chinese hamster cells 3

G09	XRCC4	X-ray repair complementing defective repair in Chinese hamster cells 4
G10	XRCC5	X-ray repair complementing defective repair in Chinese hamster cells 5
G11	XRCC6	X-ray repair complementing defective repair in Chinese hamster cells 6
G12	XRCC6BP 1	XRCC1 binding protein 1

Supplementary Table S2

**DNA repair list**
RPA4
PMS2P2
MSH4
GTF2H4
HUS1
RPA1
PARP1
FANCC
PMS2
RAD9B
RPA2
MSH5
CHEK2
FANCE
NBN
BRCA2
ATM
BRCA1
NEIL3
RPA1
RAD51B
ERCC2
RECQL4
RAD51C
RAD1
TOP3A
WRNIP1
PARG
XPA
RRM1
MBD5
RPA3
PMS2P5
MBD3
RAD54B
N4BP2
UNG
MUTYH
RAD51D
PARP3
NEIL2
RAD50
EXO1

FANCA
BRIP1
SMUG1
POLQ
PMS2
PCNA
PNKP
MGMT
POLB
POLE3
RAD23B
RAD52
RAD23A
APEX1
TOP2A
MSH2
ERCC4
XPG
LIG1
FANCG
POLE2
LIG4
XRCC4
BLM
TP53
PMS1
POLD2
ALKBH2
OGG1
PRKDC
ATP23
NTHL1
XRCC5
GTF2H3
ERCC1
POLH
RAD54L
TOP1
LIG3
XRCC1
XRCC6
REV3L
POLD1
FAN1
POLA1
XPC

NEIL1
FEN1
TDP1
DCLRE1A
FANCM
MBD1
RAD21
RAD51AP1
ATR
MRE11
UNG
RFC1
RBBP8
TP53BP1
CDK7
PMS2P3
PARP4
RAD51
XAB2
HUS1
RAD23A
FANCF
CSNK1D
POLD3
POLE4
POLN
ERCC6
RAD9A
MBD2
RECQL5
ENDOV
TREX2
MLH1

Supplementary Table S3: Patient demographics and pathological features in ovarian cancer cohort.

		Number	Percentages
Pathology			
	Serous cystadenocarcinoma	178	53.9%
	Endometrioid	44	13.2%
	Clear cell carcinoma	24	7.2%
	Mucinous cystadenocarcinoma	46	13.8%
	Others	15	4.5%
	Mixed	17	5.1%
	missing	7	2.3%
Grade			
	1	46	13.8%
	2	60	18%
	3	171	51.5%
	missing	54	16.7%
Residual tumors			
	None/Microscopic	205	61.7%
	<1cm	34	10.2%
	>1-2 cm	15	4.5%
	>2cm	40	12%
	Missing	37	11.6%
FIGO Stage			
	I	123	37%
	II	49	14.7%
	III	128	38.5%
	IV	11	3.3%
	Missing	20	6.5%
Chemotherapy			
	Carboplatin monotherapy	100	30%
	Carboplatin + Paclitaxel	110	33%
	Other regimens *	26	8%
	No chemo	23	7 %
	unknown	72	22%
Platinum sensitivity			
	Sensitive **	250	75.6%
	Resistant	26	7.8%
	Unknown	55	16.6%
Relapse status			
	Progression-free ***	169	50.9%
	Progressed/relapsed	118	35.6%
	Unknown	44	13.3%

Other Regimens included: 3= CAP (Cyclophosphamide, Adriamycin and Cisplatin) 4 = ICON5 Trial 5 = SOCTROC Trial 6 = Carboplatin and Endoxan. 7 = Chlorambucil. For the

unknown category, the majority received platinum-based chemo but because the exact regimen was not mentioned clearly in the database, they were considered as unknown. However, for these cases the platinum sensitivity status were clearly mentioned / recorded.
**Platinum resistance was defined as patients who had progression during first-line platinum chemotherapy or relapse within 6 months after completion of platinum treatment.
*** Progression-free survival was calculated from the date of the initial surgery to disease progression or from the date of the initial surgery to the last date known to be progression-free at the end of follow-up. All the patients' dates of disease recurrence were recorded in the main sheet where we used to perform the analysis.

SUPPLEMENTARY FIGURES

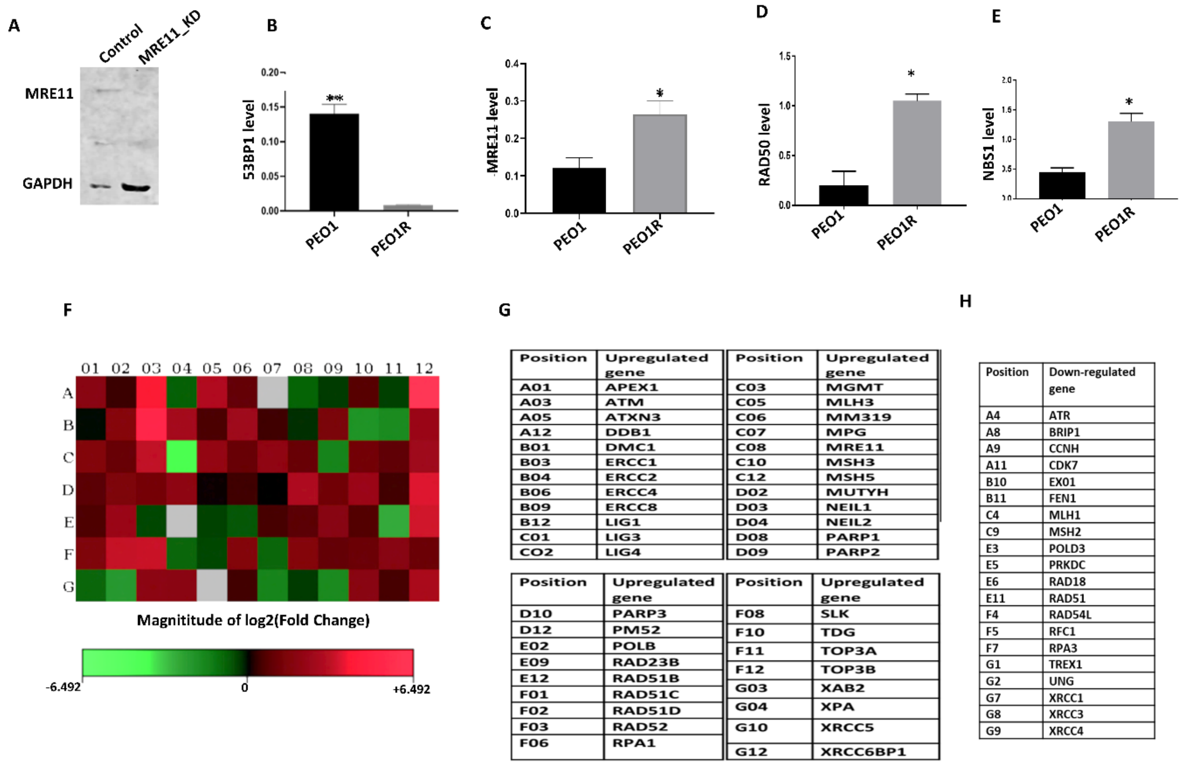

G

Position	Upregulated gene	Position	Upregulated gene
A01	APEX1	C03	MGMT
A03	ATM	C05	MLH3
A05	ATXN3	C06	MM319
A12	DDB1	C07	MPG
B01	DMC1	C08	MRE11
B03	ERCC1	C10	MSH3
B04	ERCC2	C12	MSH5
B06	ERCC4	D02	MUTYH
B09	ERCC8	D03	NEIL1
B12	LIG1	D04	NEIL2
C01	LIG3	D08	PARP1
CO2	LIG4	D09	PARP2

Position	Upregulated gene	Position	Upregulated gene
D10	PARP3	F08	SLK
D12	PM52	F10	TDG
E02	POLB	F11	TOP3A
E09	RAD23B	F12	TOP3B
E12	RAD51B	G03	XAB2
F01	RAD51C	G04	XPA
F02	RAD51D	G10	XRCC5
F03	RAD52	G12	XRCC6BP1
F06	RPA1		

H

Position	Down-regulated gene
A4	ATR
A8	BRIP1
A9	CCNH
A11	CDK7
B10	EX01
B11	FEN1
C4	MLH1
C9	MSH2
E3	POLD3
E5	PRKDC
E6	RAD18
E11	RAD51
F4	RAD54L
F5	RFC1
F7	RPA3
G1	TREX1
G2	UNG
G7	XRCC1
G8	XRCC3
G9	XRCC4

Figure S1: Protein quantification. (**A**) Quantification of MRE11 levels by western blot. (**B**) Quantification of 53BP1 levels by western blot in PEO1and PEO1R cells. (**C**) Quantification of MRE11 levels by western blot in PEO1and PEO1R cells. **(D)** Quantification of RAD50 levels by western blot in PEO1and PEO1R cells. **(E)** Quantification of RAD50 levels by western blot in PEO1and PEO1R cells. (**F**) Heat map showing DNA repair genes expression by RT2 profiler in PEO1 and PEO1R cells. (**G**) List of up-regulated genes is shown here. (**H**) List of down regulated genes is shown here.

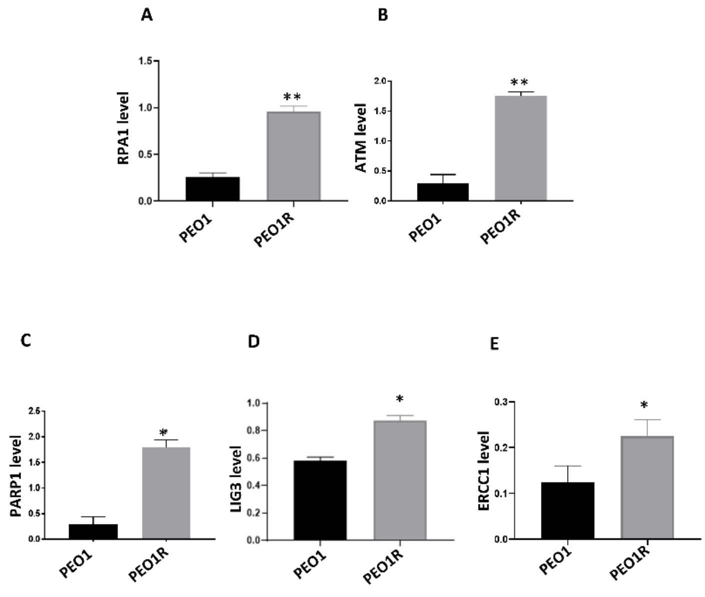

Figure S2: Protein quantification. (**A**) Quantification of RPA1 levels by western blot in PEO1 and PEO1R cells. (**B)** Quantification of ATM levels by western blot in PEO1 and PEO1R cells. (**C**) Quantification of PARP1 levels by western blot in PEO1 and PEO1R cells. (**D)** Quantification of LIG3 levels by western blot in PEO1 and PEO1R cells. (**E**) Quantification of ERCC1 levels by western blot in PEO1 and PEO1R cells. (**F)** Quantification of RAD50 levels by western blot in PEO1R and PEO1R_MRE11_KD cells. (**G)** Quantification of NBS1 levels by western blot in PEO1R and PEO1R_MRE11_KD cells.

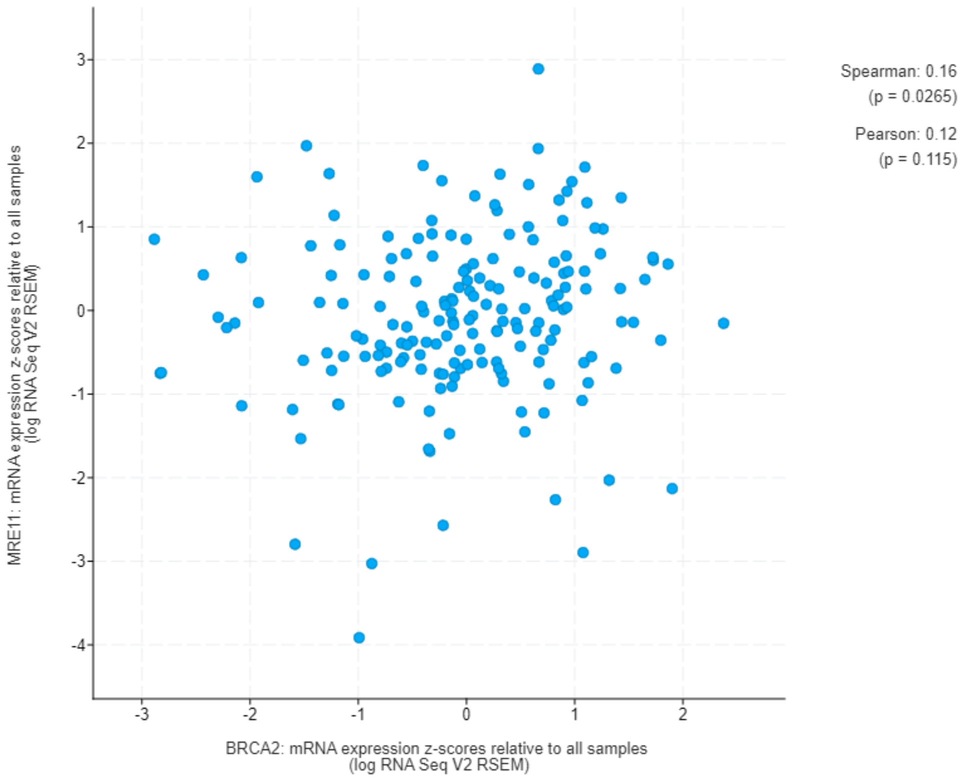

Figure S3. Correlation of *MRE11* and *BRCA2* mRNA levels in TCGA ovarian serous cystadenocarcinoma cohort (182 samples). Comparison used the log-transformed mRNA expression z-scores compared to the expression distribution of all samples (RNA Seq V2 RSEM).
